# Versatile SMAD2 and SMAD3 epitope–tagged mouse models for genomic profiling of TGFβ signaling: Uncovering GDF9–SMAD2/3 targets

**DOI:** 10.1073/pnas.2600071123

**Published:** 2026-03-30

**Authors:** Zian Liao, Qian Zhang, Keisuke Shimada, Kaori Nozawa, Suni Tang, Masahito Ikawa, Diana Monsivais, Martin M. Matzuk

**Affiliations:** ^a^Department of Pathology and Immunology, Baylor College of Medicine, Houston, TX 77030; ^b^Department of Molecular and Human Genetics, Baylor College of Medicine, Houston, TX 77030; ^c^Graduate Program of Genetics and Genomics, Baylor College of Medicine, Houston, TX 77030; ^d^Center for Drug Discovery, Baylor College of Medicine, Houston, TX 77030; ^e^Research Institute for Microbial Diseases, University of Osaka, Osaka 565-0871, Japan

**Keywords:** oocyte secreted factors, GDF9, TGFβ, SMADs, granulosa cells

## Abstract

Transforming growth factor β (TGFβ) signaling pathways are integral for a plethora of biological processes. SMAD2 and SMAD3 are the principal transcriptional effectors of TGFβ superfamily ligands, yet quantitative, genome-wide mapping of their DNA-associated complexes under physiological contexts has remained limited due to the lack of specific, robust models. Here, we generated two versatile epitope–tagged mouse models in which endogenous SMAD2 and SMAD3 proteins are globally tagged with hemagglutinin (HA) and podoplanin (PA) sequences, respectively, enabling high-fidelity profiling of SMAD2 and SMAD3 binding across tissues. To demonstrate the broad application of our models, we exemplified the usage of our lines in ovarian biology, where we defined the transcriptional programs downstream of GDF9, a key oocyte-derived ligand in folliculogenesis from the TGFβ superfamily. By integrating genomic and transcriptomic analyses, we identified direct genes induced by the GDF9-SMAD2/3 axis and identified gene sets suppressed by this signaling cascade, highlighting a previously underappreciated role of GDF9 in attenuating competing pathways to ensure proper ovarian granulosa cell fate transitions. Short-term GDF9 stimulation shifts SMAD2/3 cofactor recruitment toward lineage- and differentiation-associated transcription factors, without significant global changes in H3K27ac landscapes, indicating that GDF9 signals through targeted SMAD recruitment to preacetylated chromatin regions. Network analyses further demonstrated that GDF9-SMAD2/3 direct targets align with luteinizing hormone-driven preovulatory signaling. Together, our study generated epitope-tagged mouse models that provide extensive and applicable in vivo genetic toolkits for tissue-specific dissection of TGFβ family signaling and reveal a comprehensive, direct transcriptional network through which GDF9 coordinates granulosa cell differentiation and follicular maturation.

## Significance

Our study reports the generation and characterization of two mouse models in which endogenous SMAD2 and SMAD3 are epitope-tagged, enabling high-resolution, genome-wide mapping of SMAD2/3 complexes under physiological conditions. These models provide key in vivo tools to dissect ligand-specific transcriptional mechanisms across the TGFβ superfamily. Applying these tools to oocyte-derived GDF9 signaling in granulosa cells, we uncovered a set of direct GDF9-SMAD2/3 target genes that coordinate extracellular matrix remodeling and cell-fate transitions, while simultaneously suppressing competing signaling pathways. Our research advances the field by establishing a broadly applicable genetic toolkit for TGFβ signaling studies and revealing previously unrecognized transcriptional programs critical for ovarian function and female fertility.

Transforming growth factor β (TGFβ) signaling pathways are well-conserved, govern embryonic patterning, tissue homeostasis, immune regulation, and tissue repair, and mediate their effects through receptor-activated SMAD2 and SMAD3, which partner with SMAD4 and lineage-specific cofactors to remodel chromatin and reprogram transcription (1). Despite the essentiality of these pathways, the in vivo mechanisms by which SMAD2/3 selectively bind target loci and integrate with coregulators remain incompletely resolved, in part because native SMAD antibodies vary in specificity, sensitivity, and lot-to-lot performance, limiting reproducible genome-wide mapping (2–4). These setbacks necessitate the establishment of robust epitope-tagged *Smad2* and *Smad3* knock-in mouse lines to enable high-stringency in vivo assays across tissues and states without overexpression artifacts.

Through genetic studies in mice, sheep, and humans, our team and others have shown that TGFβ family signaling pathways direct numerous physiological processes in the ovary (5–7). Ovarian function is central to female reproductive health, determining not only fertility but also playing a key role in hormone regulation and production. Within the ovary, the growth, development, and maturation of the oocytes are tightly regulated by their bidirectional communication with the surrounding somatic granulosa cells (8–11). Such bidirectional interactions are achieved through both paracrine secretions and junctional communications, which allow the delivery of regulatory signals from the granulosa cells to regulate oocyte development during meiosis (12, 13); similarly, oocyte-secreted factors (OSFs) are also key in directing the differentiation and maintaining the cellular functions of the surrounding somatic cells (10).

Among the few discovered OSFs, members of the TGFβ superfamily have emerged as the most potent proteins in the oocyte-to-somatic cell direction of the regulatory loop. Specifically, growth differentiation factor 9 (GDF9) and bone morphogenetic protein 15 (BMP15) are the first identified OSFs that proved the active roles of the oocyte itself in organizing the ovarian follicle development, given that previous studies only considered oocytes as the recipients of the somatic–oocyte relationship (9, 14–16). Since its first functional characterization in 1996 (15, 17), GDF9 has been studied using animal models that are genetically engineered to be GDF9-deficient or harbor naturally occurring GDF9 mutations. In mice, sheep, and pigs, perturbation of GDF9 halts folliculogenesis and results in sterility (15, 18, 19). In *Gdf9* null mice, folliculogenesis is arrested at the primary follicle stage. Ovaries from these mice are small in size and lack mature follicles or corpora lutea, and anovulation is observed, leading to complete sterility (15). In Belclare sheep, heterozygous GDF9 mutations (*GDF9*^+/−^) lead to increased ovulation rate and high fecundity, resulting in dizygotic twinning or multiple births, whereas ewes with homozygous mutations (*GDF9*^−/−^) have ovarian hypoplasia, leading to follicular arrest and infertility, similar to mice (19). In pigs, *GDF9*^−/−^ gilts present a defective estrus cycle and large ovarian cysts; follicles of these gilts are arrested in the preantral stages, similar to the phenotypes observed in mice (18). In mice, *Gdf9* expression begins early in follicle development, and GDF9 protein is secreted by oocytes starting at the primary follicle stage, with sustained production through secondary and antral stages. This temporally restricted yet continuous secretion pattern, positions GDF9 as a key regulator that coordinates granulosa cell fate decisions throughout follicular maturation (20).

In humans, mutations of *GDF9* have been reported to be implicated in a range of female reproductive disorders, including infertility, polycystic ovary syndrome (PCOS), and primary ovarian insufficiency (POI) (21–25). As in sheep, mutations introducing premature stop codons or missense variants in the pro- or mature region of GDF9 have been implicated in dizygotic twinning and the subsequent development of POI. These mutations may lead to an increased number of dominant follicles per cycle, potentially accelerating follicular depletion and contributing to early exhaustion of the ovarian reserve (26–28).

Using BMP15 knockout and recombinant BMP15 protein, we demonstrated that mouse BMP15 is not as critical as GDF9 (29, 30). However, BMP15 plays a similar critical role as GDF9 in sheep and humans (31); our group showed that GDF9:BMP15 heterodimers are the most potent oocyte TGFβ ligand (10, 30). Granulosa cells from *Gdf9* null mouse ovaries exhibit elevated inhibin α expression, and excess inhibin subsequently contributes to follicular arrest, as additional genetic removal of inhibin α (*Inha*^−/−^;*Gdf9*^−/−^) permits follicles to progress to the multilaminar stage, albeit with abnormal structure and nonfunctional theca cell differentiation (32–34). These studies of single- and double-mutant mice indicate that GDF9 and activins act in a sequential and nonredundant manner during folliculogenesis, with GDF9 restraining early activin-driven granulosa cell overgrowth, while later follicle development is dominated by activin signaling that promotes granulosa proliferation at the expense of oocyte growth (7).

The signaling cascade of GDF9 starts with the receptor complex activation, composed of TGFβ type 1 (ALK5 or ALK4) and type 2 (BMPR2, ACVR2A, or ACVR2B) receptors. The receptor complex gets phosphorylated upon ligand binding and subsequently phosphorylates downstream effector proteins, SMAD2 and SMAD3 (R-SMADs) to initiate intracellular signaling. SMAD2/3 form homo-oligomers upon phosphorylation, together with SMAD4 (co-SMAD), translocate to the nucleus to direct gene transcription (30). Given the potent roles of GDF9 in ovarian biology, researchers have been heavily invested in understanding the application potential of GDF9 in optimizing the clinical approaches for women with infertility or ovarian dysfunctions (35). However, the comprehensive list of direct transcriptional targets of GDF9 remain poorly defined. Here, we generated two physiologically relevant mouse models in which the endogenous SMAD2 and SMAD3 proteins are epitope-tagged with HA (hemagglutinin) and PA (podoplanin) tags, respectively. Utilizing these mouse models, our goal was to precisely map the SMAD2/3-DNA interactions across tissues, particularly within ovarian granulosa cells, through genomic profiling. By coupling the genomic data with transcriptomic analyses, we aimed to systematically characterize the direct GDF9-responsive genes. This strategy would allow us to elucidate the molecular circuitry underlying GDF9 signaling during folliculogenesis and uncover therapeutic targets to overcome ovarian dysfunctions.

## Results

### Generation of Mouse Models with Globally Tagged SMAD2 and SMAD3 Proteins.

**Fig. 1.**
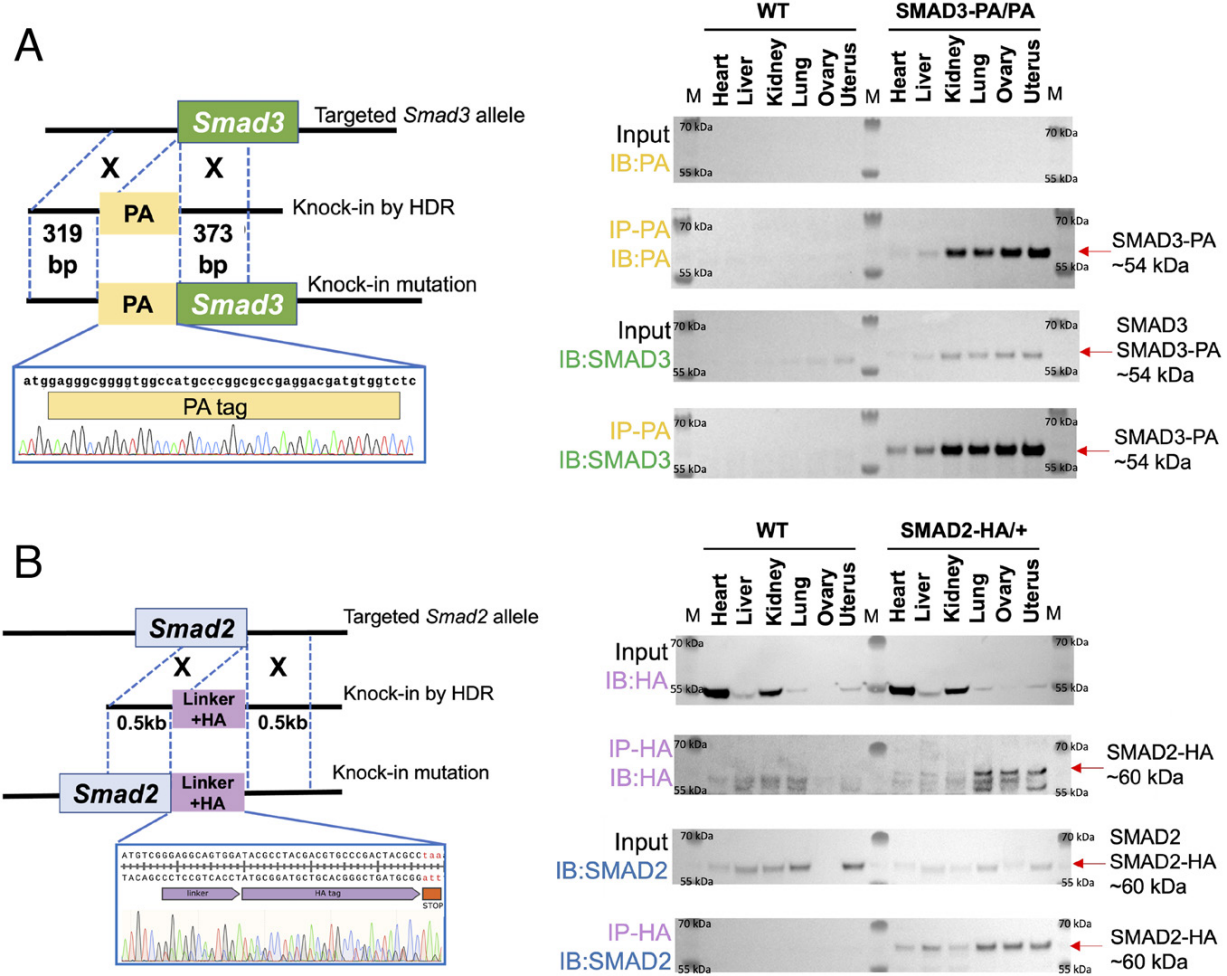
Genetically engineered mouse lines with global HA-tagged SMAD2 and PA-tagged SMAD3 proteins. (*A* and *B*) *Left*: Schematic approaches for generating N-terminal PA-tagged *Smad3*^PA/PA^ and C-terminal HA-tagged *Smad2*^HA/+^ and knock-in mouse lines. Sanger sequencing results confirming the correct insertions are included in the zoom-in windows. *Right*: Western blot analysis demonstrating the immunoprecipitation of PA-tagged SMAD3 and HA-tagged SMAD2 proteins from tissue panels from the tagged mouse lines. Tissues from wild-type (WT) mice were used as negative controls. M in the Western blot analysis represents the marker for protein size ladder.

The GDF9 signaling axis has been established as one of the requirements for the expansion of granulosa cells (36). SMAD2/3 have been demonstrated to be downstream effectors of GDF9 signaling pathways to regulate granulosa cell-specific gene expression (30, 37). Despite the critical roles of GDF9 signaling, a comprehensive map of direct SMAD2/3 target genes is lacking, motivating us to delineate the roles of SMAD2 and SMAD3 in oocyte-somatic cell communication. We aimed to delineate the roles of SMAD2 and SMAD3 in oocyte-somatic communication. We applied the CRISPR approach to generate genetically engineered knock-in mice that harbor an HA-tagged *Smad2* allele (herein called *Smad2*^HA/+^) and a PA-tagged *Smad3* allele (herein called *Smad3*^PA/PA^) (Fig. 1 *A* and *B*). The HA tag and the PA tag were inserted into the C-terminus of the SMAD2 and the N terminus of the SMAD3 proteins, respectively. The successful insertions were visualized by the genotyping results shown in *SI Appendix*, Fig. S1*A*, and we confirmed the correct genomic insertion by Sanger sequencing (Fig. 1 *A* and *B*). To ensure the tagged proteins can be detected, we performed immunoprecipitation followed by western blot analysis of the heart, liver, kidney, lung, ovary, and uterine tissues from *Smad2*^HA/+^ and *Smad3*^PA/PA^ mice, and we confirmed that the HA-tagged SMAD2 and PA-tagged SMAD3 proteins can be readily detected by HA and PA antibodies (Fig. 1 *A* and *B*). Molecular sizes and the protein expression patterns of the HA antibody-detected SMAD2 protein and the PA antibody-detected SMAD3 protein were comparable to those of the SMAD2 antibody-detected SMAD2 protein and the SMAD3 antibody-detected SMAD3 protein, respectively (Fig. 1 *A* and *B*). Although the heterozygous HA-tagged SMAD2 mice (*Smad2*^HA/+^) are healthy and viable, we were unable to generate homozygous HA-tagged SMAD2 mice (*Smad2*^HA/HA^) due to potential embryonic lethality. (*SI Appendix*, Fig. S1*B*). We used heterozygous intercrosses of *Smad2*^HA/+^ mice to generate the homozygous *Smad2*^HA/HA^ genotype. Across two independent mating trials encompassing a total of 10 litters, genotyping of all offspring revealed the presence of 35 *Smad2*^HA/+^ heterozygous and 24 wild-type (WT) pups, but no *Smad2*^HA/HA^ homozygous pups were recovered. The observed genotype distribution deviated from the expected Mendelian ratio for a viable homozygous allele, indicating that the *Smad2*^HA/HA^ genotype cannot survive to birth. This outcome is consistent with the known nonredundant requirement for SMAD2 in early embryonic patterning, as SMAD2 but not SMAD3 is essential for lineage specification and anterior–posterior axis formation (38, 39). This homozygotic lethality further suggests that SMAD2 function is highly dosage- and sequence-sensitive, such that even subtle modification of the endogenous locus may be incompatible with embryogenesis and/or later development. In summary, we successfully generated and utilized viable mouse models with global heterozygous HA-tagged SMAD2 and homozygous PA-tagged SMAD3 alleles for our studies.

### GDF9 Stimulation of Granulosa Cells Leads to Transcriptomic Changes Facilitating Ovarian Functions.

**Fig. 2.**
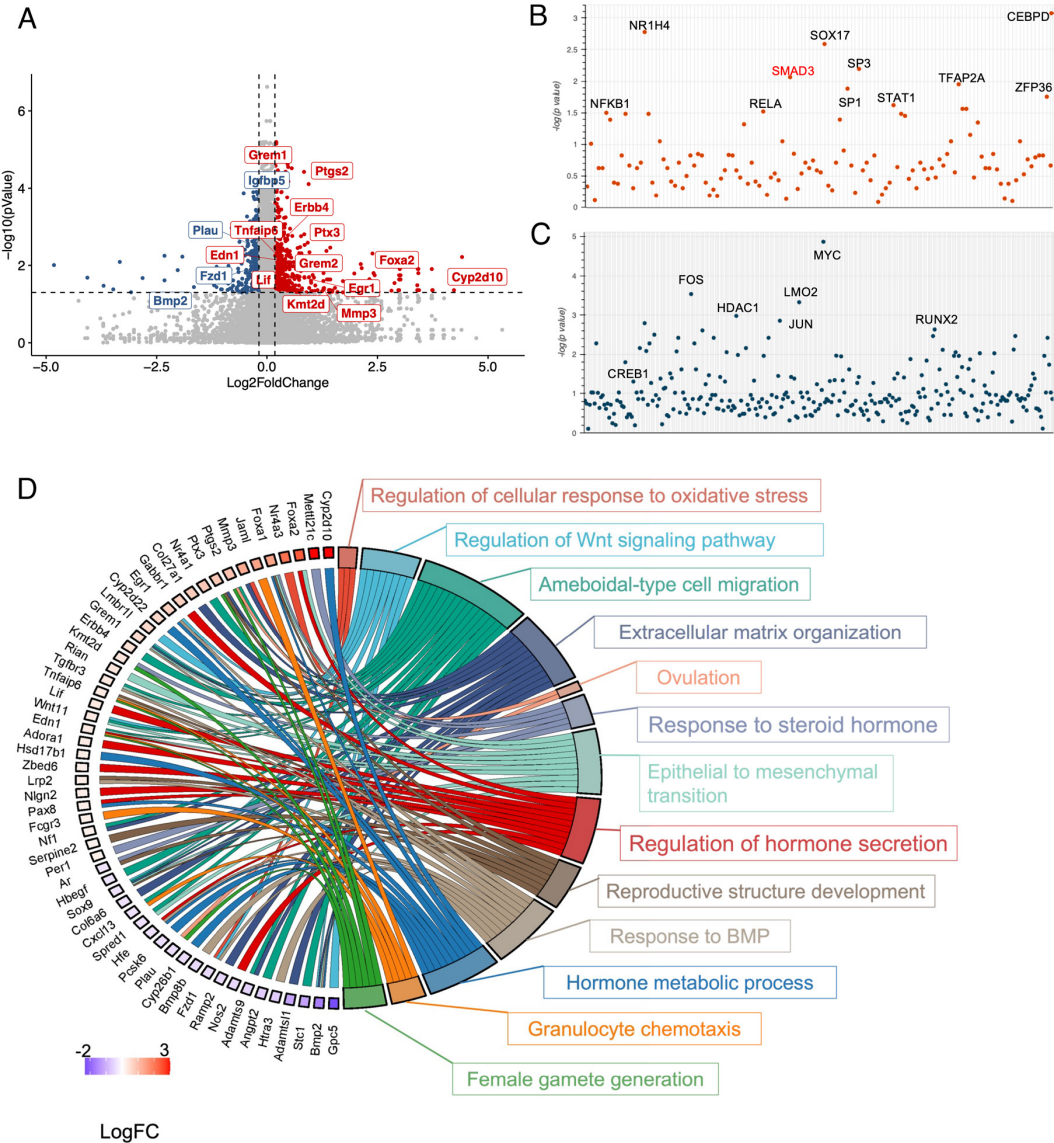
Transcriptomic profiling of the mouse granulosa cells upon GDF9 treatment. (*A*) Volcano plot of the differentially expressed genes from the RNA-seq analysis. Red color indicates up-regulated genes while blue color indicates down-regulated genes. (*B* and *C*) Results from Transcriptional Regulatory Relationships Unraveled by Sentence-based Text Mining (TRRUST) based transcription factor enrichment analysis on the up-regulated (*B*) and down-regulated (*C*) genes. (*D*) Cord plot of the Gene Ontology enrichment results from the differentially expressed genes.

To investigate the transcriptomic changes induced by GDF9 exposure in mouse granulosa cells, we collected granulosa cells from preovulatory follicles in pregnant mare serum gonadotropin (PMSG)-stimulated 21 to 28-d-old mice. The primary mouse granulosa cells from SMAD3^PA/PA^ mice were then stimulated with recombinant mouse GDF9 (100 ng/mL) for 4 h and performed bulk RNA-sequencing. Differential gene expression analysis revealed a total of 857 genes significantly altered in response to GDF9 stimulation (|LogFC| > 0.18, adjusted *P* value < 0.05) compared to control treatment, with 530 genes upregulated and 327 genes downregulated (Fig. 2*A*). Among the up-regulated genes, we validated a series of known GDF9 target genes, including *Ptgs2*, (LogFC = 0.94), *Ptx3* (LogFC = 0.85), *Has2* (LogFC = 1.08), and *Tnfaip6* (LogFC = 0.35), all of which have been shown to be integral in supporting cumulus expansion by modulating hyaluronic acid matrix formation (40, 41). In addition to the canonical GDF9-responsive genes, our analysis revealed a number of transcripts not previously associated with GDF9 signaling. Notably, *Mirlet7e*—a member of the highly expressed let-7 microRNA family in granulosa cells—was significantly upregulated. The let-7 family has been extensively implicated in regulating folliculogenesis, oocyte maturation, and granulosa cell expansion, underscoring its potential role as a downstream effector of GDF9 in ovarian function (42–44). The complete gene expression profile results are included in Dataset 1.

To further deduce the upstream transcriptional regulators potentially driving the observed gene expression patterns, we performed Transcriptional Regulatory Relationships Unraveled by Sentence-based Text Mining (TRRUST) based transcription factor (TF) enrichment analysis on the differentially expressed genes (45) (Fig. 2 *B* and *C*). From the up-regulated gene sets, transcription factors activating cellular growth, differentiation, and steroidogenesis were enriched (Fig. 2*B*). Notably, SMAD proteins, consistent with their known involvement in GDF9-mediated signaling, were among the top-ranked predicted regulators. Additional enriched TFs included CEBPD, RELA/NFKB, TFAP2A, and SOX17.

To investigate potential transcriptional repressors in mediating gene down-regulation in response to GDF9, we also examined the enriched transcription factors associated with down-regulated genes. Several known transcriptional repressors and chromatin modifiers were identified, including HDAC1 and HDAC5, which are known to compact chromatin and silence gene expression (46), suggesting a potential role for epigenetic regulation in the GDF9-mediated gene network. Additional enriched repressors included ZBTB7A and ZBTB16, which are zinc finger proteins with established roles in gene silencing and lineage specification (47, 48), and SIRT1, a NADº-dependent deacetylase involved in stress adaptation and transcriptional repression in granulosa cells (49, 50). Furthermore, CREB1, known to be involved in follicular growth, ovulation, and ovarian disease (51), may also help suppress alternative differentiation programs, thereby reinforcing a granulosa cell-specific fate. Collectively, these findings suggest that GDF9 not only activates gene networks that are critical for folliculogenesis but also actively represses competing transcriptional programs through specific transcription factors and epigenetic regulators. The complete enrichment results are listed in the Dataset 2. Next, we performed Gene Ontology (GO) analysis on both up- and down-regulated differentially expressed genes with a focus on the biological process enrichment. As shown in Fig. 2*D*, we highlight the enrichment results that included categories related to ovarian functions (ovulation; response to steroid hormone; regulation of hormone secretion and hormone metabolic process), the BMP/WNT signaling pathways, extracellular organization, and reproductive structure development. The complete GO enrichment results are listed in the Dataset 3.

Together, these results indicate that GDF9 induced a distinct and functionally unique transcriptional program in granulosa cells, characterized by pro-survival, pro-differentiation, and oocyte-supportive gene networks. This transcriptomic pattern extends beyond canonical targets and involves additional pathways that contribute to folliculogenesis and oocyte maturation, thereby highlighting the central role of GDF9 in coordinating follicular development and ovulatory competence.

### Genomic Profiling of SMAD2 and SMAD3 Reveals Direct Transcriptional Regulations upon GDF9 Stimulation.

To define the direct transcriptional regulatory landscape in response to GDF9 stimulation, we performed cleavage under targets and release using nuclease (CUT&RUN) sequencing on the granulosa cells treated with recombinant mouse GDF9 from the *Smad3*^PA/PA^ mice. Given the canonical roles of SMAD2/3/4 complexes in transducing the GDF9 signaling, we utilized SMAD2, PA, and SMAD4 antibodies to map DNA binding activities of the SMAD2/3/4 in the granulosa cells derived from the *Smad3*^PA/PA^ tagged mouse. Meanwhile, to examine whether GDF9 has a role in modulating transcriptionally active regions, we used H3K27ac as a marker to map the regulatory landscape (enhancers and promoters) in the granulosa cells.

**Fig. 3.**
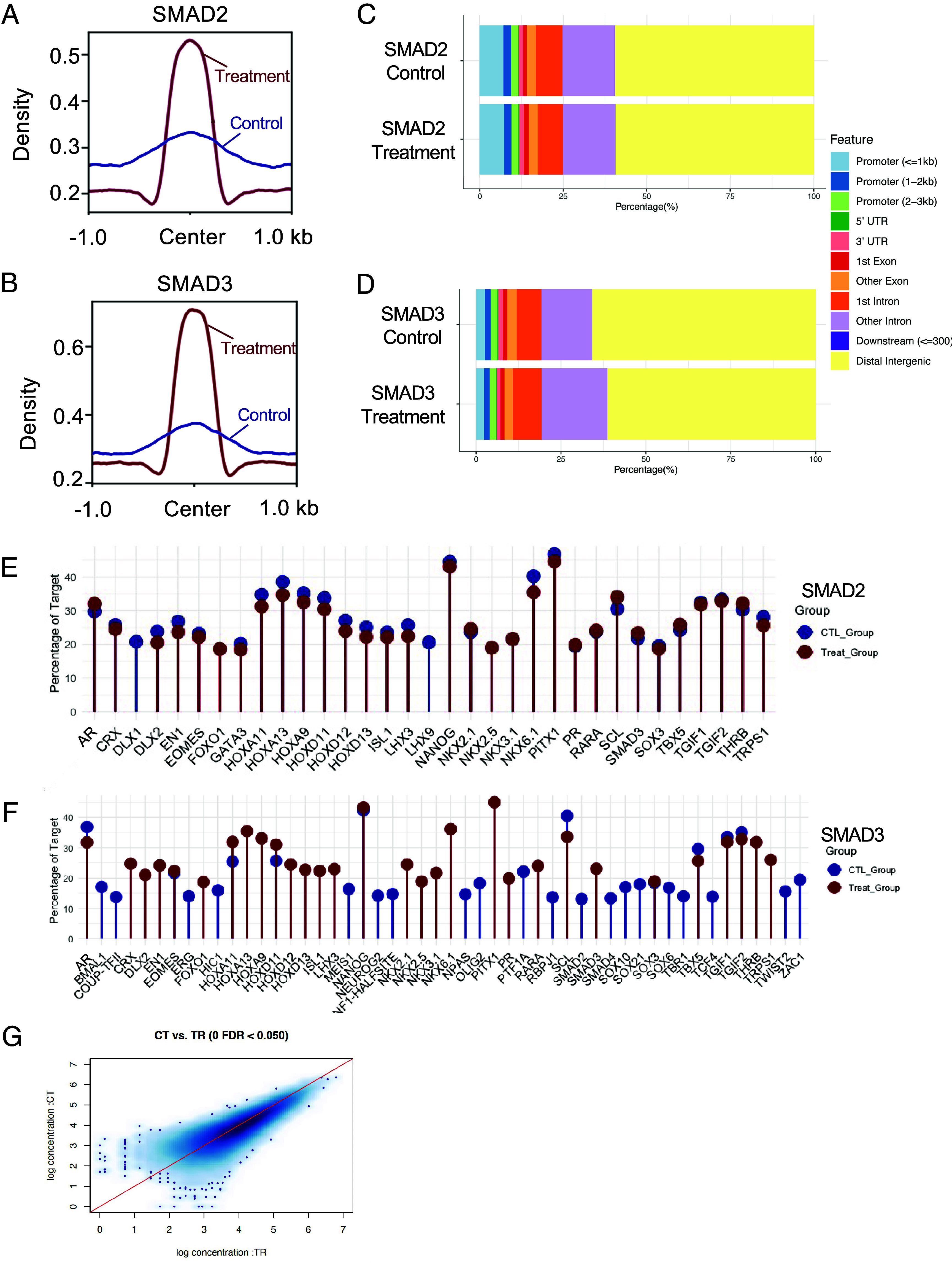
Genomic profiling of SMAD2 and SMAD3 binding activities in mouse granulosa cells. (*A* and *B*) Peak density plots of SMAD2 and SMAD3 in both control and GDF9-treated groups, one biological replicate was used for this exemplary plot. (*C* and *D*) Consolidated bar plot showing the distribution of annotated peaks from SMAD2 and SMAD3 under both control (Control) or GDF9-treated (Treatment) conditions. (*E* and *F*) Motif enrichment results from the SMAD2 (*F*) and SMAD3 (*G*) groups. CTL_Group represents the control group, while Treat_Group represents the GDF9-treated group. (*G*) Mean-Average plot showing the normalized signal intensity for H3K27ac marks between the control (CT) and the GDF9-treated (TR) conditions.

The fragment length distribution of immunoprecipitated DNA has a characteristic enrichment at ~100 bp, consistent with the transcription factor target binding pattern (52). Similarly, in the H3K27ac groups, the fragment lengths showed an enrichment between 150 and 200 bp, corresponding to the mononucleosomal deposition, indicating high-quality chromatin immunoprecipitation (*SI Appendix*, Fig. S2). Next, we generated peak density profiles of SMAD2 and SMAD3 in both control and GDF9-treated groups. GDF9 treatment yielded a marked summit-centered enrichment compared with controls, indicating increased SMAD2/3 chromatin occupancy consistent with pathway activation (Fig. 3 *A* and *B*). We annotated high-confidence peaks using ChIPseeker (53). In the SMAD2 group, under the control condition, 9.41% peaks are mapped to the promoter regions (±3 kb of promoter) (Fig. 3*C*). Upon GDF9 stimulation, the distribution of the SMAD2 peak remains largely unchanged, with 9.53% of the peaks falling within the promoter regions. In the SMAD3 group, 8.19% and 5.76% of the peaks are mapped to the promoter region in the control and GDF9 treatment groups, respectively (Fig. 3*D*). The full list of the annotated peaks is included in the Datasets 4–7. De novo motif profiling of SMAD2 and SMAD3 CUT&RUN peaks revealed condition-specific cofactor signatures (Fig. 3 *E* and *F*). For SMAD2, the overall landscape was similar between the control and treatment groups. The treated peaks showed enrichment in homeobox and NKX family motifs (e.g., HOXA9/11/13, HOXD11/12/13, NKX2-1/5, NKX3-1), in addition to the canonical SMAD motif. Notably, PITX1 and NANOG motifs were among the most prevalent, of which the interaction between SMAD proteins has been previously proposed/validated in other contexts (54, 55). In contrast, SMAD3 displayed a more pronounced cofactor shifting: in addition to both being enriched for SMAD motifs, GDF9-treated peaks were preferentially enriched for lineage/developmental motifs, including homeobox modules (HOXA9/11/13 and HOXD11/12/13), NKX factors (NKX2.1, NKX2.5, NKX3.1), compared with controls. Among peaks from the control group, enriched motifs showed a higher representation of TGIF1/2 (corepressor-associated), SOX family (SOX6/10/21), consistent with a more SMAD-centric, repressive/cofactor-limited context at baseline (56–59). These findings indicate that GDF9 drives SMAD2/3 interactions with transcription-activation-centric modules to initiate context-specific transcriptional programs.

Genome-wide H3K27ac profiles between the control and GDF9 groups were highly concordant. Differential analysis detected no significantly differentially acetylated regions (Fig. 3*G*). With robust library QC metrics (fragment-length mononucleosome mode, duplication rate) matched, the likelihood of technical confounders is minimized. Notably, prior studies have shown that chromatin accessibility and enhancer acetylation can change rapidly, often within 1 to 2 h of stimulation, when signaling induces global chromatin accessibility remodeling, indicating that our 4-h exposure is sufficient to detect such effects if present (60, 61). The absence of detectable H3K27ac changes at this time point therefore suggests that downstream transcriptional effects driven by GDF9 are mediated primarily through targeted SMAD2/3 recruitment to preexisting active chromatin, rather than through widespread remodeling of the chromatin accessibility landscape.

**Fig. 4.**
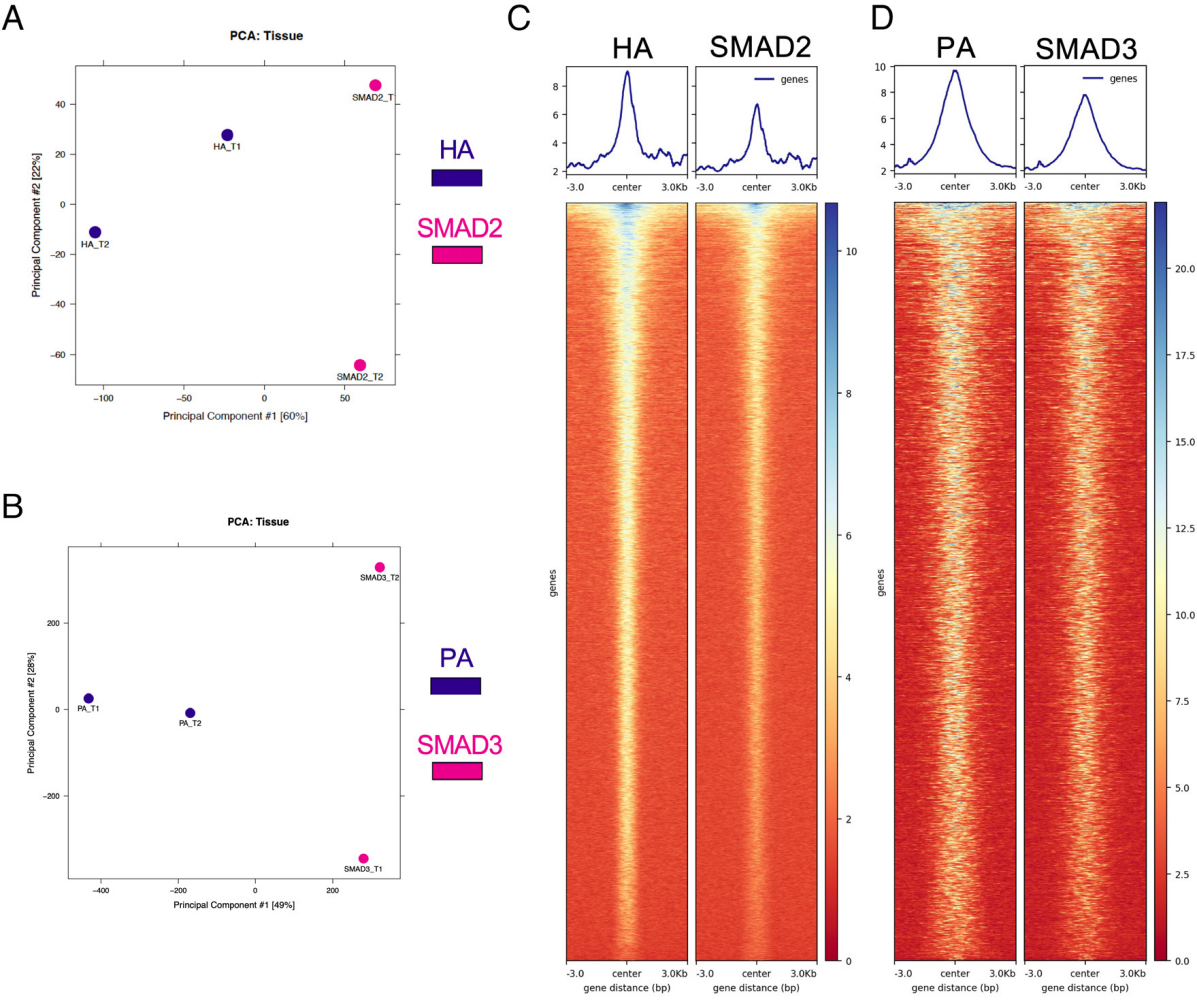
Systematic characterizations of SMAD2, HA, SMAD3, and PA marks. (*A*) Principal component analysis plot of the CUT&RUN results from SMAD2 and HA antibodies under the GDF9 treatment condition. (*B*) Principal component analysis plot of the CUT&RUN results from SMAD3 and PA antibodies under the GDF9 treatment condition. (*C*) Peak density plot of CUT&RUN results from SMAD2 and HA antibodies under the GDF9 treatment condition. (*D*) Peak density plot of CUT&RUN results from SMAD3 and PA antibodies under the GDF9 treatment condition.

We next compared the performance of a native SMAD2 antibody with an anti-HA antibody for mapping genomic SMAD2 binding using the *Smad2*^HA/+^ epitope–tagged line. The two assays identified largely overlapping target sets, with 83.14% and 82.44% of binding genes shared under control and GDF9-stimulated conditions, respectively (Fig. 4*A* and Dataset 4), indicating highly concordant SMAD2 occupancy. We performed a similar comparison for SMAD3 using native SMAD3 and anti-PA antibodies, observing that 77.15% (control) and 69.17% (GDF9-stimulated) of bound genes were shared between the two antibodies (Fig. 4*B* and Dataset 5). Notably, the anti-HA antibody consistently yielded higher peak densities, resulting in increased normalized read depth and an improved signal-to-noise ratio at shared sites (Fig. 4*C*). The anti-PA antibody likewise demonstrated a modestly stronger signal relative to the SMAD3 antibody (Fig. 4*D*). Collectively, these data validate the HA- and PA-tagged approaches as robust and complementary strategies for quantitative, genome-wide mapping of endogenous SMAD2 and SMAD3, as well as for downstream integrative analyses.

### Direct Target Genes of GDF9-SMAD2/3 Axis Direct the Physiological Transformation of Granulosa Cells During Oocyte Maturation.

**Fig. 5.**
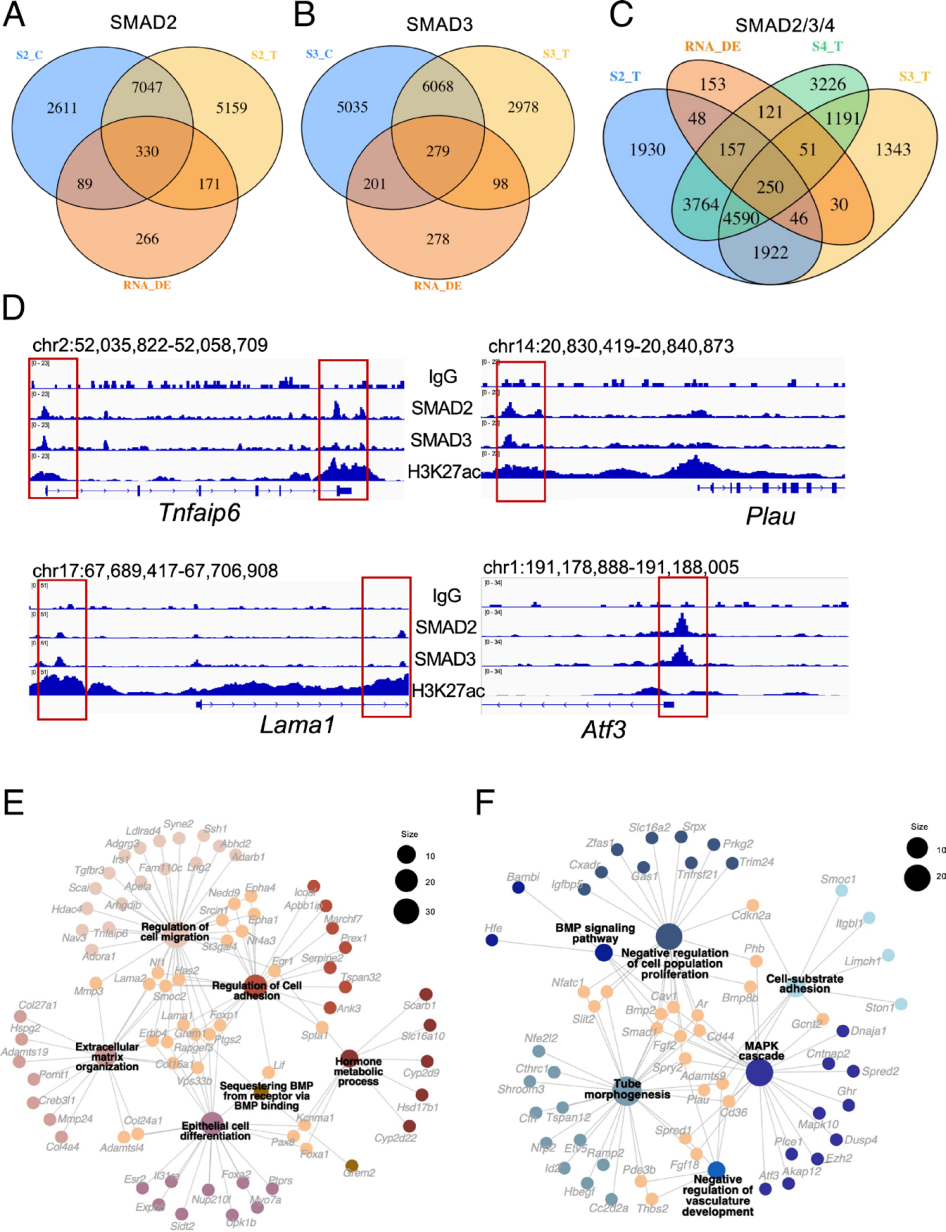
Identification of the direct target genes of the GDF9-SMAD2/3 signaling axis. (*A*–*C*) Venn diagrams showing the shared and unique genes bound by SMAD2 (*A*), SMAD3 (*B*), and SMAD4 (*C*). S2 represents SMAD2 groups, S3 represents SMAD3 groups, S4 represents SMAD4 groups. _C represents the control condition, and _T represents the GDF9 treatment conditions. RNA_DE represents the genes that are differentially expressed from the RNA-seq results. (*D*) Integrative Genomics Viewer (IGV) track view of the exemplary loci of the direct targets of SMAD2 and SMAD3, H3K27ac denotes the active chromatin regions. Genomic coordinates labeled correspond to the mm10 genome. (*E* and *F*) Gene-concept network plot visualizing the core up-regulated target genes (*E*) and down-regulated target genes (*F*) identified in our analysis. Circle packing nodes represent enriched categories linked to target genes, with node size corresponding to the number of genes associated with each category.

To define direct GDF9-SMAD2/3 targets in mouse granulosa cells, we integrated our matched RNA-seq with SMAD2/3/4 CUT&RUN maps (Datasets 6–11). First, we intersected differentially expressed genes after GDF9 stimulation with genes bearing SMAD2 or SMAD3 peaks (within promoter ±3 kb, UTR, exon or first intron), respectively (Fig. 5 *A* and *B*). This yielded 590 SMAD2-associated and 578 SMAD3-associated direct targets, indicating broad, ligand-responsive SMAD occupancy at the indicated loci. We then aimed to refine the high-confidence direct targets by requiring that gene candidates i) are differentially expressed upon GDF9 stimulation and ii) harbor a SMAD2 or SMAD3 peak within regulatory intervals (promoter ±3 kb, UTR, exon or first intron). This integrative filter produced a concise set of direct targets (Fig. 5*C* and Dataset 12), comprising 349 core up-regulated and 233 core down-regulated genes. Genome browser (IGV) tracks illustrate two established GDF9 targets-TNF α induced protein 6 (*Tnfaip6*) (up-regulated) and urokinase plasminogen activator (*Plau*) (down-regulated) with dense SMAD2/3 CUT&RUN signal. We also highlight two additionally nominated direct targets, *Lama2* and *Atf*, drawn from the core up-regulated and core down-regulated sets, respectively (Fig. 5*D*).

Gene-Concept Network plot analysis of the core GDF9-regulated genes revealed coherent, pathway-level rewiring (Fig. 5 *E* and *F*). Among the up-regulated core set (Fig. 5*E*), genes converged on extracellular matrix organization, regulation of cell adhesion/migration, and epithelial cell differentiation, with additional enrichment for hormone metabolic processes and sequestering BMP from receptors. Central connectors included classic cumulus-expansion genes [e.g., hyaluronan synthase 2 (*Has2*), prostaglandin-endoperoxide synthase 2 (*Ptgs2*), and *Tnfaip6*] (30, 36, 40). and matrix/adhesion components (e.g., *Lama1/2*, *Col2a1*/*Col24a1*, and *Adamts* family), which encode basal-lamina components and ECM proteases that sculpt the perioocyte matrix (62–65), echoing a model of GDF9-driven microenvironmental remodeling in granulosa cells. In contrast, the down-regulated core set (Fig. 5*F*) mapped to MAPK cascade, BMP signaling, cell-substrate adhesion, tube morphogenesis, and negative regulation of cell-population proliferation, with hubs such as *Spry2*, *Plau*, *Fgf2*, and *Bmp2*. Functionally, *Fgf2/Spry2* dampens receptor-tyrosine-kinase/MAPK signaling; its repression is consistent with granulosa cell differentiation programs (66). *Plau* promotes extracellular matrix proteolysis; lowering *Plau* would stabilize the pericumulus matrix during the granulosa cell expansion (40, 67). Finally, *Bmp2* as a direct down-regulated target suggests attenuation of SMAD1/5/9 cross-talk, emphasizing the signaling balance between SMAD2/3 versus SMAD1/5 that drives the dedicated transcriptional state in granulosa cells (36). These network analyses indicate that GDF9 activates gene programs that promote matrix assembly and adhesive remodeling while attenuating MAPK/BMP signaling cascades and suppressing proliferation circuits, aligning with the observed SMAD2/3 binding shifts and the functional transition of granulosa cells during follicular maturation.

### GDF9-SMAD2/3 Direct Targets are Enriched in the Granulosa Cells and Align with Luteinizing Hormone-driven Preovulatory Signaling.

**Fig. 6.**
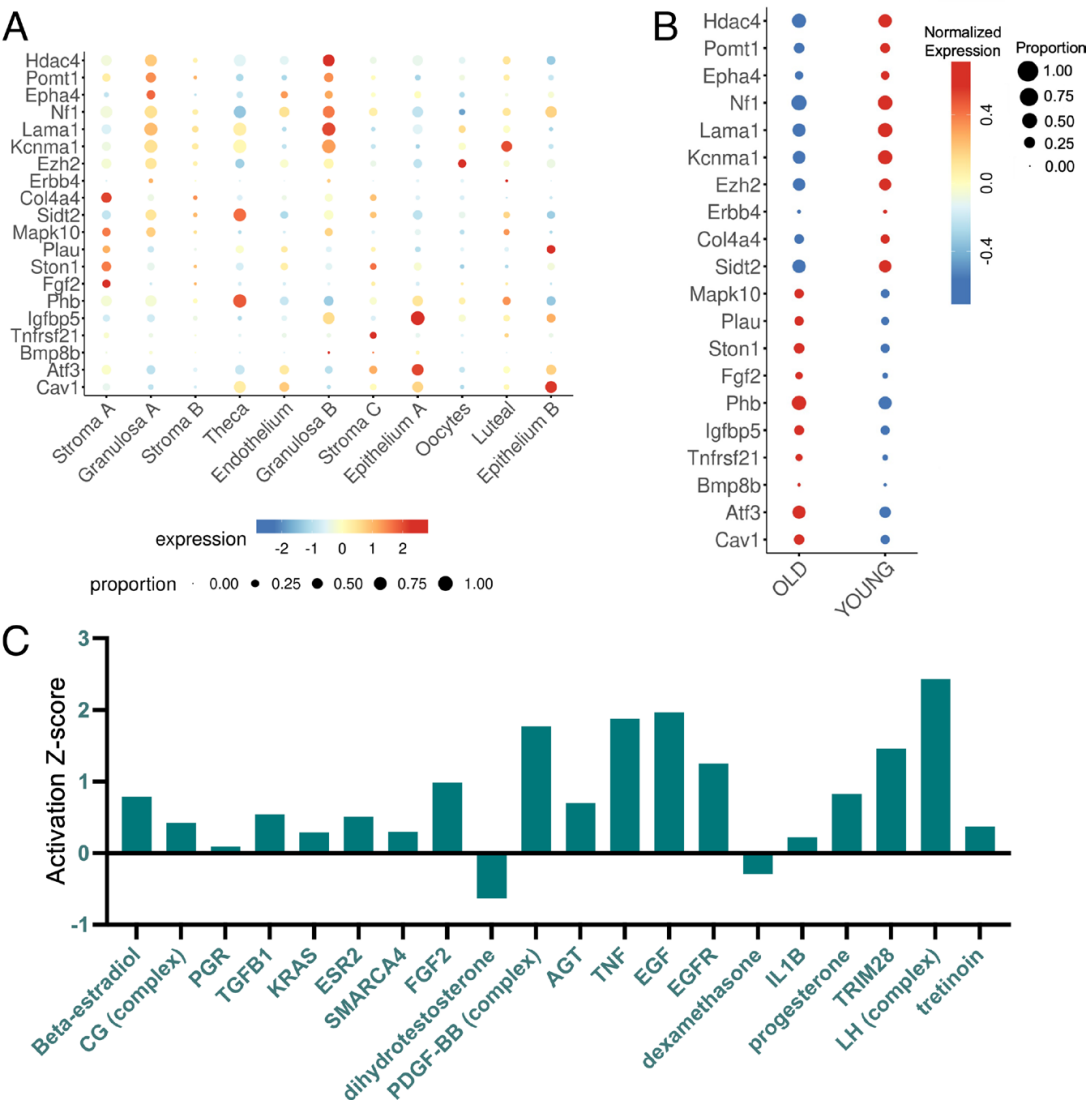
Expression patterns of the core-direct target genes in the mouse ovary. (*A*) Bubble plot visualizing the expression levels of the direct target panel across different cell types in the mouse ovary. Dot size represents the percentage of cells expressing each respective gene. Dot color represents the averaged expression levels. Single-cell RNA-seq data were accessed through the GEO deposition of GSE232309, with cell types annotated as previously described (68). (*B*) Bubble plot visualizing the expression levels of the direct target panel from the old and young mouse ovary. (*C*) Visualization of the upstream regulator analysis in the Ingenuity Pathway Analysis. The Activation z-score indicates the predicted activation (positive value) or inhibition (negative value) relationship.

To contextualize our direct targets in vivo, we projected the core GDF9-SMAD2/3 gene sets onto a published single-cell ovary atlas (68) and analyzed the expression patterns across cell types. (Dataset 12) The up-regulated direct targets discussed here (e.g., *Hdac4*, *Pomt1*, *Lama1*, *Kcnma1*) were selectively enriched in the granulosa cluster, which is consistent with our cellular input and the cell type upon which GDF9 signaling is biologically active (Fig. 6*A*). Age-stratified analysis in the young (3-mo-old) and old (9-mo-old) showed that the core-up-regulated genes have higher expression levels in young ovaries and are blunted in older ovaries, whereas the core-down-regulated direct-target set displayed the opposite pattern, increasing with age (Fig. 6*B*). These trends suggest that ovarian aging is accompanied by a rebalancing away from GDF9-driven matrix/adhesion programs toward MAPK/BMP and proteolytic cues, potentially contributing to impaired cumulus expansion, suboptimal oocyte competence, and altered follicle dynamics. To further address whether age-associated differences in GDF9-SMAD2/3 target gene expression reflect changes in cellular composition rather than general transcriptional attenuation, we examined changes in cell percentages across age groups within the previously annotated granulosa cell subclusters (68). In addition to increased average expression in older ovaries, several core GDF9-down-regulated targets [e.g., *Plau* or mitogen-activated protein kinase 10 (*Mapk10*)] exhibited an increase in the proportion of antral and preantral granulosa cells with age, as visualized by reduced dot sizes and diminished cell-percentage heatmap signal (*SI Appendix*, Fig. S3). These findings indicate that ovarian aging is accompanied not only by global dampening of GDF9-responsive gene expression (69) but also by shifts in emphasis to antral and preantral granulosa cells engaging this transcriptional program, consistent with emerging models of age-related cellular heterogeneity (70). Together, these data support a dual mechanism whereby aging alters both the magnitude of GDF9-SMAD2/3 signaling output and the cellular composition distribution within granulosa cell populations, potentially contributing to compromised follicular support functions.

To infer the upstream transcriptional cues that may cooperate with or oppose the GDF9-SMAD2/3 signaling axis, we performed upstream regulator analysis of the GDF9-SMAD2/3 core-direct target genes using the Ingenuity Pathway Analysis tool. Our results predicted a shift toward gonadotropin and growth-factor signaling (Fig. 6*C*). Notably, luteinizing hormone (LH) emerged as the strongest predicted activator (highest activation Z-score), consistent with the timing of GDF9 action during the preovulatory window (71). Additional strong predicted activators included EGF/EGFR, TNF, and PDGF-BB, suggesting that GDF9-SMAD2/3 transcriptional outputs converge with growth factor and inflammatory remodeling inputs to support follicular maturation, ECM remodeling, and cumulus expansion (72, 73). Additionally, a more moderate enrichment toward beta-estradiol/ESR2, progesterone/PGR, TGFβ1, FGF2, AGT, SMARCA4, and TRIM28 was observed. In contrast, dihydrotestosterone and dexamethasone scored negatively, indicating the predicted inhibitory effect of androgen and glucocorticoid for the GDF9-driven transcriptional changes.

Collectively, these predictions are consistent with a pro-ovulatory, pro-cell differentiation environment: GDF9-induced gene programs align with LH pathway activation and ECM/adhesion remodeling, while anti-inflammatory/glucocorticoid and androgenic inputs can attenuate such process. This regulatory signature supports the model that GDF9 engages SMAD2/3 while cooperating with gonadotropin functions to drive granulosa-cell differentiation and cumulus expansion.

## Discussion

The SMAD2 and SMAD3 transcription factors are the signal transduction hub for the versatile signals emitted by TGFβ superfamily members (74). Ligands, including TGFβs, activins, BMP15, and specifically GDF9, which was investigated in this study, preferentially signal through SMAD2/3 complexes (74). While structurally similar, SMAD2 and SMAD3 have distinct roles in various biological processes [i.e., embryonic development (75)] and disease contexts [i.e., cancers (76)]. To overcome long-standing technical limitations in mapping specific SMAD2 and SMAD3 genomic occupancy, we generated knock-in mouse models harboring endogenous epitope tags in the SMAD2 (HA tag) and SMAD3 (PA tag) loci. These mouse lines enable a stable and universal platform for high-fidelity dissection of TGFβ superfamily biology across tissues and physiological states. Compared to native SMAD2 antibodies, our HA-tagged SMAD2 model enables highly consistent immunoprecipitation results using a well-characterized HA antibody, yielding a substantially stronger signal-to-noise ratio and cleaner backgrounds in the CUT&RUN profiling (Fig. 4 *C* and *D*). In addition, the HA-tagged SMAD2 and PA-tagged SMAD3 mouse lines can improve detection sensitivity for weak or transient binding, without the issue of structural masking (i.e., conformational changes, posttranslational modifications). Our endogenous tagging approach also circumvents the overexpression artifacts that introduce complicated stoichiometry or dosage effects. Collectively, these advantages make our tagged mouse models a next-generation reference platform, enhancing our ability to reveal previously unrecognized SMAD2/3 regulatory functions with precise spatial and temporal resolution.

In our study, we integrated RNA-seq results with SMAD2/3 genomic profiling to define a high-confidence catalog of direct GDF9 targets, exemplifying the potential usage of our mouse models in ovarian biology studies. This catalog includes canonical cumulus-expansion genes (e.g., *Has2*, *Ptgs2*, *Tnfaip6*, *Ptx3*) and additionally nominated candidates (e.g., *Lama1*, *Col4a4*, *Pomt1*, *Adamtsl4*) that are actively transcribed in granulosa cells. These genes converge on extracellular matrix assembly, adhesion remodeling, and cell migration, which are essential for ovarian follicle growth, granulosa cell cumulus expansion, and ovulation. The expanded target set refines the transcriptional wiring downstream of GDF9 and highlights previously underappreciated cofactors that are positioned to modulate granulosa cell transformation and improve follicular competence. Our data support the model in which oocyte-derived GDF9 triggers SMAD2/3 translocation into the nucleus and binding at preacetylated regulatory elements in granulosa cells, thereby activating ECM/adhesion gene programs.

Our results not only identified direct genes that are activated by the GDF9-SMAD2/3 axis, but also uncovered another major functional aspect of GDF9 signaling: coordinated suppression of defined transcriptional programs. While GDF9 is classically explored as a ligand that activates gene expression programs during ovarian follicle maturation, we demonstrate that GDF9 signaling through SMAD2/3 can also directly attenuate gene classes linked to MAPK/BMP pathways, cell-substrate adhesion, and negative regulation of vasculature development. These suppressed gene cohorts suggest that, like other ligands in the TGFβ superfamily (77), GDF9 not only induces transcription but also participates in suppressing signaling “noise” that would otherwise derail the transformation of granulosa cells. These data also provide orthogonal evidence how hyperandrogenism suppresses follicular development through a GDF9-driven program (78). Specifically, the identification of *Bmp2* as a direct transcriptional target suppressed by acute GDF9-SMAD2/3 signaling again underscores the negative feedback through selective repression of parallel signaling inputs. This regulatory logic echoes the concept of signaling wiring described for BMP4, in which negative feedback restricts signaling range to ensure robust developmental outcomes (79). In the ovarian follicle, such cross-regulatory control may be particularly important during granulosa cell fate transitions, where competing TGFβ superfamily cues must be precisely coordinated. Such a dual-functioning mechanism is conceptually consistent with the model in which the oocyte tightly directs the granulosa cell states during the periovulatory window.

The lack of global H3K27ac changes indicates that GDF9-SMAD2/3 signaling after 4-h incubation of cells with recombinant GDF9 does not broadly open chromatin; instead, its transcriptional output likely hinges on recruiting specific cofactors (i.e., lineage factors and/or nuclear receptors) to enhancer and promoter regions. This dependency points to actionable hypotheses: perturbing cofactor availability or binding pockets could precisely tune GDF9 responses without introducing global changes, suggesting potential therapeutic options for follicular dysfunction. Consistent with this, the age-associated patterns that we observed in the single-cell datasets of the GDF9-SMAD2/3 direct targets imply that weakening of the oocyte-granulosa circuit may contribute to declining oocyte quality with age. Future studies that investigate whether modulating SMAD2/3 cofactors or boosting oocyte-derived cues in aging follicles are warranted to help advance ovarian health.

Although we successfully generated the heterozygous *Smad2*^HA/+^ mice, we observed embryonic lethality in the homozygous *Smad2*^HA/HA^ mice. These findings can be interpreted in several contexts. First, in contrast to the viability of *Smad3*-null mice reported in prior work (39, 75), *Smad2*-null mice are embryonic lethal (38), indicating nonredundant roles of the two receptor-regulated SMADs in development. In many contexts, SMAD2 can partially compensate for the loss of SMAD3 (80, 81), but SMAD3 cannot substitute for SMAD2 during early embryonic patterning and lineage specification. This asymmetric imbalance between SMAD2 and SMAD3 during development likely underlies the lethality observed with the homozygous HA-tagged *Smad2* allele. Second, although the HA allele is functionally neutral, the homozygote lethality suggests that even subtle changes of the *Smad2* locus can be incompatible with embryogenesis, consistent with the known roles of SMAD2 in early lineage specification and anterior–posterior patterning (82). These findings underscore paralog-specific requirements and caution that SMAD2 is exquisitely sequence-sensitive; future experiments (e.g., insertion of alternative epitopes, phenotyping the homozygous embryos) will help dissect whether lethality reflects developmental timing, tissue specificity, or tag-induced hypomorph.

We note that the high-confidence catalog of direct GDF9 targets identified in this study reflects transcriptional responses captured under a defined experimental condition, namely, a single GDF9 concentration (100 ng/mL) and a fixed treatment duration (4 h). In vivo, GDF9 is secreted by the oocyte and diffuses across the follicle, likely establishing concentration gradients that may elicit distinct transcriptional programs in spatially or functionally heterogeneous granulosa cell subpopulations (41, 83). Future studies incorporating dose–response, temporal profiling, and spatially resolved approaches will be important to further refine how GDF9-SMAD2/3 signaling diversifies gene regulation within the follicular microenvironment.

In summary, our endogenously tagged-SMAD mouse lines enable quantitative, tissue-specific multimodal mapping of SMAD2 and SMAD3 complexes across development and in disease settings, providing a stable, yet versatile platform for transcriptomic, genomic, and proteomic assays in all tissue types. These models provided a robust foundation to dissect pathway crosstalk; for example, with MAPK, BMP, and/or nuclear-receptor signaling using genomics, proteomics, and pharmacologic approaches. Our integrative genomics analysis emphasized that SMAD2 and SMAD3 are core effectors of GDF9 signals in granulosa cells and can reprogram extracellular-matrix and adhesion circuits while attenuating competing pathways. The tagged lines generated in our study deliver a versatile genetic toolkit to interrogate and ultimately manipulate TGFβ superfamily signaling pathways in many organ systems, and in the proof-of-concept case herein, we have been able to further understand the oocyte-granulosa crosstalk that underpins ovarian function, with potential translational avenues: prioritizing cofactor dependencies as therapeutic entry points, refining biomarkers of follicle competence, and guiding targeted interventions to restore follicular deficiencies.

## Materials and Methods

### Generation of Knock-In Mouse Lines.

*Smad3*^PA/PA^ knock-in (KI) mice were generated using a similar approach previously described (84). We used single-strand DNA (ssDNA) that contained a PA tag (42 bp) with left (319 bp) and right (373 bp) homology arms. The crRNA that targets regions close to the start codon, tracrRNA, Cas9 ribonucleoprotein complex, and ssDNA were injected into fertilized eggs obtained by B6D2F1 × B6D2F1 mating (Fig. 1*A*). Of the 382 fertilized eggs injected, 213 eggs were transplanted into the oviducts of pseudopregnant females. A total of 55 potential founder mice were born, and 2 pups possessed the desired mutations. Homozygous *Smad3*^PA/PA^ mice were maintained in the C57BL/6J × 129S5/SvEvBrd mixed genetic background. The sequences of the sgRNA, HDR repair oligonucleotide, and genotyping primers are provided in *SI Appendix*, Table S1.

To generate *Smad2*^HA/+^ mice, we utilized the CRISPR-EZ approach (85) with the help of the Genetically Engineered Rodent Model Core at Baylor College of Medicine. Briefly, Cas9 protein, single-guide RNA (sgRNA), and a homology-directed repair (HDR) donor carrying the HA tag and linker sequences were coelectroporated into zygotes obtained from in vitro fertilization of B6D2F1 male and female mice. (Fig. 1*B*). The resulting embryos were cultured overnight to reach the 2-cell stage, after which they were transferred into the oviducts of pseudopregnant CD-1 recipient females (Center for Comparative Medicine, Baylor College of Medicine). Offspring were subsequently screened for successful knock-in alleles by PCR using primers flanking the inserted HA tag. The sequences of the sgRNA, HDR repair oligonucleotide, and genotyping primers are provided in *SI Appendix*, Table S1.

### Animal Ethics Compliance and Tissue Collection.

Mice included in this study were maintained in a temperature-controlled vivarium (70 °F ± 2 °F and 20 to 70% relative humidity) on a 12-h light/dark cycle. All animal procedures were conducted in accordance with protocols approved by the Institutional Animal Care and Use Committee (IACUC) at Baylor College of Medicine. Experiments were performed using female mice aged 3 to 4 wk on a mixed C57BL/6J × 129S5/SvEvBrd genetic background. Euthanasia was achieved by isoflurane induction and subsequent cervical dislocation, in accordance with the approved procedures by the Baylor College of Medicine IACUC.

### Cleavage Under Targets and Release Using Nuclease (CUT&RUN) in Mouse Granulosa Cells.

To collect the granulosa cells, 3 to 4 wk old female mice were injected with 5 IU of Pregnant Mare Serum Gonadotropin (PMSG, Prospec, HOR-272) and ovaries were collected 46 h later. Granulosa cells were then purified and cultured following the previously published protocol (30, 40) for 4 d before the GDF9 treatment. The experiments were performed using pooled biological replicates from primary granulosa cells cultured from 8 mice, respectively. On the day of the GDF9 treatment, mouse GDF9 (R&D, 739-G9-010/CF) was added to the granulosa cell culturing media to reach the final concentration of 100 ng/mL. The granulosa cells were digested into single-cell suspensions 4 h after the GDF9 treatment and subsequently processed using the CUT&RUN protocol essentially as described (52, 86). The GDF9 concentration (100 ng/mL) and treatment duration (4 h) were selected based on a previous publication (30) and preliminary optimization experiments (*SI Appendix*, Fig. S4), in which the minimal dose and shortest exposure sufficient to induce the phosphorylation of SMAD2/3 and increase the canonical COX2 protein expression were determined. Approximately 55,000 cells were used for each reaction. Concanavalin A-coated magnetic beads (Bangs Laboratories, BP531) were conjugated to the cells, and the resulting bead–cell complexes were resuspended in 100 μL of Antibody Buffer [recipe is described in Ref (86)] per reaction. Immunoprecipitation was initiated by adding 1 μg of the indicated antibodies: IgG control (Sigma, I5006), anti-HA (EpiCypher, 13-2010), anti-PA (Fujifilm, NZ-1), or anti-SMAD2 (Abcam, ab33875). After overnight incubation at 4 °C, the bead–cell complexes were washed and subsequently incubated with pAG-MNase (EpiCypher, 15-1016) at room temperature for 10 min. Bead–cell–pAG-MNase complexes were washed and chromatin digestion was initiated by adding 1 μL of 100 mM CaCl_2_ to each reaction, followed by incubation at 4 °C for 2 h. Reactions were terminated by adding 50 μL of Stop Buffer [recipe is described in Ref (86)] and incubating at 37 °C for 10 min. The supernatant was collected, and DNA was purified by phenol–chloroform extraction and ethanol precipitation. Sequencing libraries were prepared using the NEBNext Ultra II DNA Library Prep Kit (New England BioLabs, E7645) according to the manufacturer’s instructions, and paired-end 150 bp sequencing was performed on an Illumina NovaSeq X Plus platform.

### Bioinformatic Analysis for CUT&RUN Data and Reanalysis of Published Single-Cell RNA Seq Data.

Data processing for CUT&RUN dataset largely follows the previously published pipeline (52, 86) with minor modifications. In short, after demultiplexing and quality control, clean reads were mapped to the mouse genome mm10 and the *E. coli* genome U00096.3 (for Spike-in) by Bowtie2. Uniquely mapped reads were kept for the Spike-in normalization. The normalization was done by multiplying primary genome coverage by a scale factor (100000 / fragments mapped to the *E. coli* genome). CUT&RUN peaks were called using SECAR (87) with the default parameters. Track visualization was done by bedGraphToBigWig (88), and Integrative Genomics Viewer (IGV) was used for visualization. Common peaks between replicates were identified with the “mergePeaks” function in HOMER v4.11 (89). ChIPseeker (90) was used to apply annotation to the identified peaks. HOMER v4.11 with findMotifsGenome.pl with the genome of mm10 and default parameters (89) was used to perform motif enrichment. Peak heatmaps and the density plots were generated using deepTools 2.4.2. To analyze gene expression patterns in different ovarian cell types, we accessed previously published mouse ovarian single-cell RNA-seq data with cell type reference from young (3-mo) and old (9-mo) mice, available from GSE232309. Sequencing data quality control and filtering criteria were as previously described (68). *Amh* was used as the distinguishing marker for the granulosa cell types. The normalized bubble plots were generated by averaging the expression, which was log-transformed and then plotted.

### RNA-seq Sample Preparation and Data Analysis.

Total RNA from mouse granulosa cells was extracted using the QIAGEN RNeasy kit (QIAGEN, Cat #74104) following the manufacturer’s protocol. Poly-A-enriched, strand-specific libraries were generated using the NEBNext Ultra II Directional RNA Library Prep Kit (NEB, E7760S, E7490S) with 500 ng input RNA following the manufacturer’s protocol. Libraries were sequenced, targeting 30 million read pairs per sample. Demultiplexed FASTQ files were generated with bcl2fastq v2.20. Read QC and preprocessing were performed as previously described (91). Alignment and quantification were done using STAR v2.7.11b against mouse genome mm10 (92). Aligned reads were assigned to genes with featureCounts v2.1.1. Count matrices were analyzed in R with DESeq2 v1.48.2 (93).

### Western Blot Analysis of Immunoprecipitation (IP-WB).

IP-WB process largely follows the previously published pipeline (86) with minor modifications. Pulverized tissues were lysed in NETN buffer (20 mM Tris-HCl pH 8.0, 150 mM NaCl, 0.5 mM EDTA, 10% glycerol, 0.5% NP-40), and 1.5 mg of total lysate was used per IP. Lysates were incubated with anti-HA (Cell Signaling, C29F4) or anti-PA (Fujifilm, NZ-1) antibodies for 1 h at 4 °C, followed by the addition of protein G magnetic beads (Thermo Fisher, 88847) for an additional 1 h. Beads were washed five times with NETN buffer, then denatured for gel electrophoresis. Samples were resolved on 4-12% Bis-Tris gels (Thermo Fisher, NP0321BOX), transferred to nitrocellulose membranes, and the membranes were then blocked with 5% milk in TBST, and incubated overnight at 4 °C with the primary antibodies anti-HA (Cell Signaling, C29F4), anti-PA (Fuji Film, NZ-1), anti-SMAD2 (Cell Signaling, D43B4), or anti-SMAD3 (Cell Signaling, C67H9) at 1:1,000 dilution. Membranes were then washed and incubated with HRP-conjugated secondary antibodies for 1 h at room temperature. Subsequently, membranes were washed again, developed, and imaged using the iBright FL1500 system.

## Supplementary Material

Appendix 01 (PDF)

## Data Availability

Sequencing data and analyses are deposited in the Gene Expression Omnibus under accession number GSE315433 (94).
