# Supplementary material for: Versatile SMAD2 and SMAD3 epitope–tagged mouse models for genomic profiling of TGFβ signaling: Uncovering GDF9–SMAD2/3 targets
Source: Proc Natl Acad Sci U S A. 2026 Mar 30;123(14):e2600071123. doi: 10.1073/pnas.2600071123 (PMC13056123; doi:10.1073/pnas.2600071123)
Supplement: Supplementary file 1 — Appendix 01 (PDF) [file pnas.2600071123.sapp.pdf]

Supporting Information for

Versatile SMAD2 and SMAD3 epitope-tagged mouse models for genomic profiling of TGFβ signaling: uncovering new GDF9-SMAD2/3 targets

Zian Liao[1,2,3,4], Qian Zhang[5, †], Keisuke Shimada[5,§], Kaori Nozawa[1,4,‡], Suni Tang[1,4], Masahito Ikawa[5], Diana Monsivais[1,4], and Martin M. Matzuk*[1,2,3,4]

[1]Department of Pathology & Immunology, Baylor College of Medicine, Houston, TX, 77030, USA
[2]Department of Molecular and Human Genetics, Baylor College of Medicine, Houston, TX, 77030, USA
[3]Graduate Program of Genetics and Genomics, Baylor College of Medicine, Houston, TX, 77030, USA
[4]Center for Drug Discovery, Baylor College of Medicine, Houston, TX, 77030, USA
[5]Research Institute for Microbial Diseases, Osaka University, Osaka, 565-0871, Japan
[†]Present address: Laboratory Animal Center, Chongqing Medical University, 401331, China
[§]Present address: Laboratory of Experimental Animal Science, School of Veterinary Medicine, Rakuno Gakuen University, Hokkaido, 069-0836, Japan.
[‡]Present address: Department of Advanced Medical Technologies, National Cerebral and Cardiovascular Center, Osaka,564-8565, Japan.

* Corresponding Author: Martin M. Matzuk, MD, PhD, Center for Drug Discovery, Department of Pathology and Immunology, Baylor College of Medicine, One Baylor Plaza, Houston, TX 77030; Email: mmatzuk@bcm.edu; Telephone: 713-798-6451

This PDF file includes:

Supplementary Figures S1 to S4
Supplementary Table 1
Supplementary Datasets 1-12

Supplementary Figure 1:

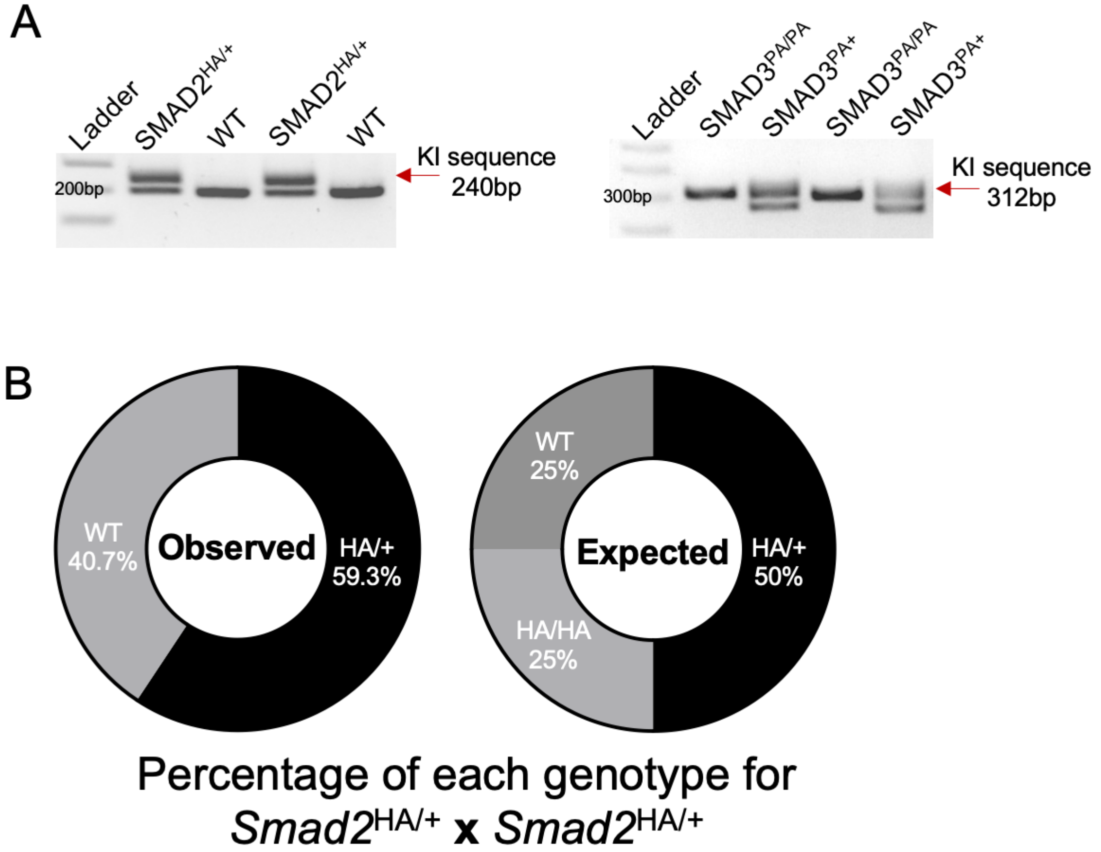

Supplementary Figure 1: Percentage of offspring genotypes of the heterozygotic mating scheme. A) Genotyping results of the tagged mouse lines. **B)** Percentage of offspring genotypes of the mating scheme of SMAD2$^{HA/+}$ x SMAD2$^{HA/+}$.

Supplementary Figure 2.

A

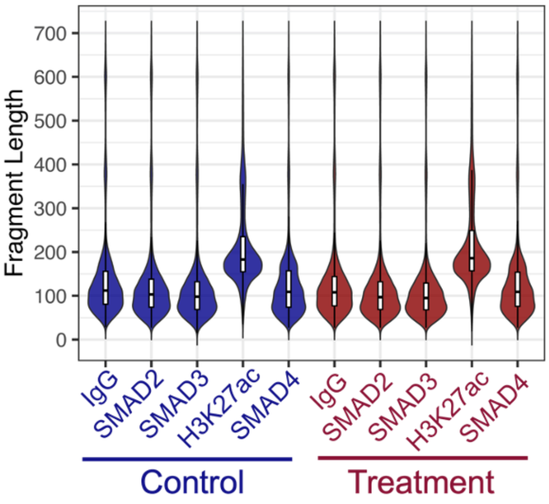

Supplementary Figure 2: **Fragment length characterization of the CUT&RUN libraries. A)** Violin plots showing the fragment length distribution of immunoprecipitated DNA for each indicated group.

Supplementary Figure 3.

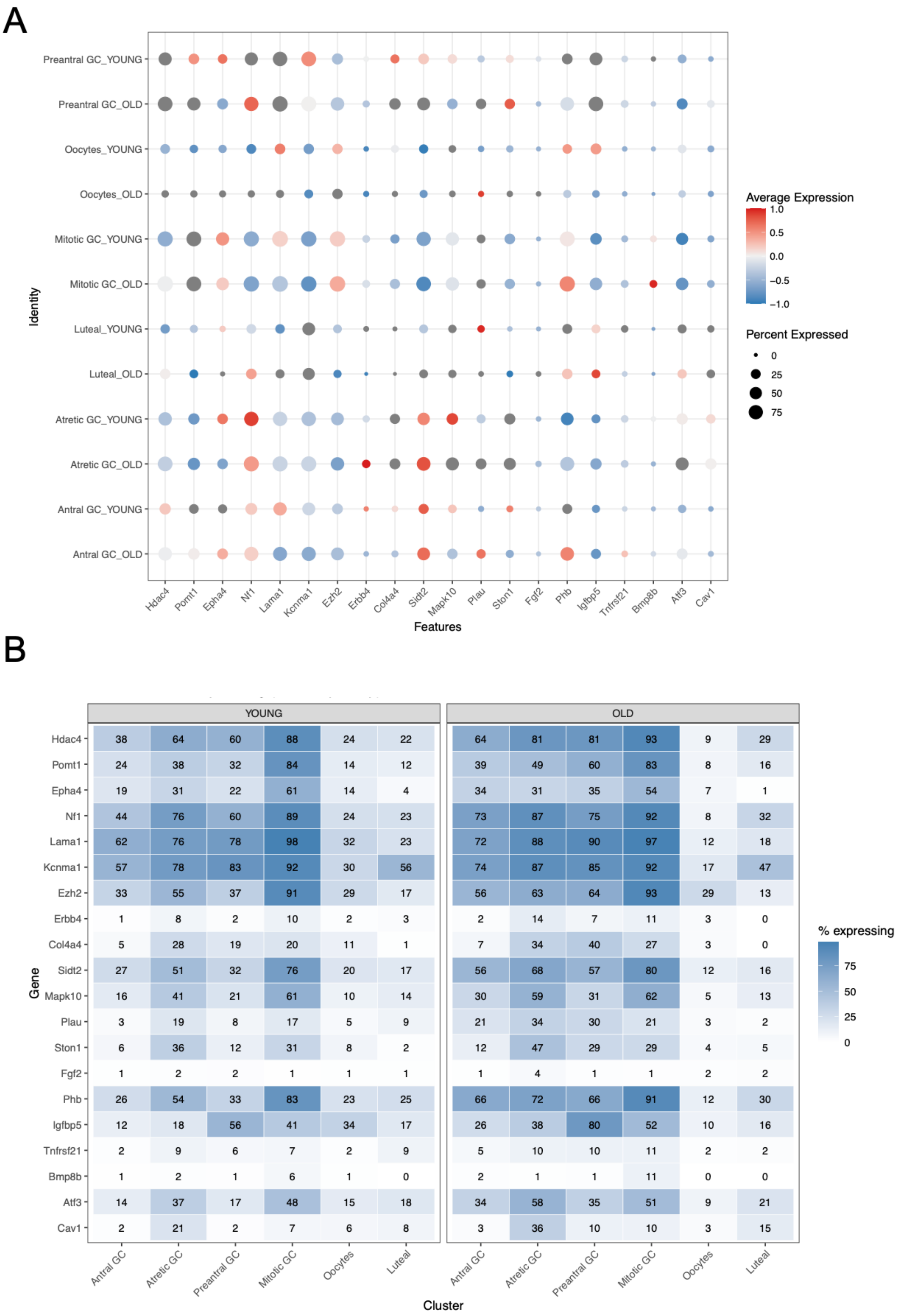

Supplementary Figure 3: **Average expression and cell percentage of GDF9 direct target genes in old and young mouse ovaries. A)** Dot plot visualizing the expression levels of the direct target panel across the “Granulosa Cells” clusters in young and old mouse ovaries, with cell types annotated as previously described[68]. Dot size represents the percentage of cells expressing each respective gene within the granulosa cell clusters. Dot color represents the averaged expression levels. Data were accessed through the GEO deposition of GSE232309. **B)** Heatmap visualizing the percentage of cells that express the direct target panel across the “Granulosa Cells” clusters in young and old mouse ovaries, with cell types annotated as previously described[68]. Data were accessed through the GEO deposition of GSE232309. GC, granulosa cell.

Supplementary Figure 4.

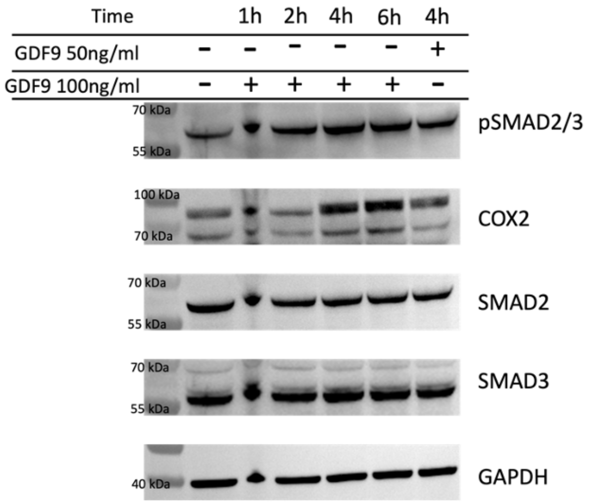

Supplementary Figure 4: Optimization of GDF9 treatment dosage and duration. Western blot analysis of phosphorylated SMAD2/3 (pSMAD2/3), COX2, total SMAD2, and total SMAD3, with GAPDH used as a loading control. Following GDF9 treatment (100 ng/mL), activation of the GDF9–SMAD2/3 axis was evident by 4 h, as indicated by increased SMAD2/3 phosphorylation and induction of the canonical downstream target COX2.

Supplementary Table:

Supplementary Table 1: DNA sequences used in the study.

Name	Sequence/Catalog number	Usage	Note
S2-F1	TCAAACAGGAGG CAGACAGT	For *Smad2*$^{HA/+}$ genotyping	
S2-R1	GCAAACAGTCCA CGGGATCA		
S3-F1	CGGGCAAGTTCT CCAGAGTT	For *Smad3*$^{PA/PA}$ genotyping	
S3-R1	AACTGCCCCGTC TTCTTGAG		
sgRNA-SMAD2	CTGTTACAGCGA GTCTTTGA TGG	sgRNA for SMAD2-HA	GRCm38:Chr18:76 304733-76304755
crRNA-SMAD3	GGCAGGATGGAC GACATGGC	crRNA for SMAD3-PA	
HDR template for SMAD2	GATTGAACTTCAT CTGAATGGCCCT CTGCAGTGGCTG GACAAAGTATTAA CTCAGATGGGAT CCCCTTCAGTGC GATGCTCAAGCA TGTCGGGAGGCA GTGGATACGCCT ACGACGTGCCCG ACTACGCCTAAA CCAATCAAAGAC TCGCTGTAACAG CTCCTCCGTCGT AGT	HDR template for generating the knock-in mice	Sequence for generating repair oligo of homology-directed repair

HDR template for SMAD3	GCGGCACCCAAA<br>CAGCTACCCCGT<br>GCGGAAACCCAA<br>ACTTTCTACTGCC<br>ACTTGGAGTCTC<br>GCGGCCGCCGC<br>CTCCGCCCCGCG<br>CGTCCGGGGCCT<br>GCCCGTCAGTCC<br>GTCGGTCCGCGT<br>GGAGCAGCTCGG<br>GCGCCGCCGTGC<br>TCCCGATCCCCG<br>CAGCTGCAGCGC<br>CGCAGTCCTGGC<br>CCGGACGCCCG<br>GGCAAGTTCTCC<br>AGAGTTAAAAGC<br>GAAGTTCGGGCG<br>AGGCGCGGGCC<br>GAGCTGCCTCTG<br>AGCGCCCCCGGC<br>GTCCCCAGTGCG<br>CCCAGCCCCGCC<br>GGGGGCGCCGG<br>TGACCCTTCGGT<br>GCCAGCCATGGA<br>GGGCGGGGTGG<br>CCATGCCCGGCG<br>CCGAGGACGATG<br>TGGTCTCGTCCA<br>TCCTGCCCTTCA<br>CCCCCCCGATCG<br>TGAAGCGCCTGC<br>TGGGTTGGAAGA<br>AGGGCGAGCAGA<br>ACGGGCAGGAG<br>GAGAAGTGGTGC<br>GAGAAGGCGGTC<br>AAGAGCTTGGTG<br>AAGAAGCTCAAG<br>AAGACGGGGCAG<br>TTGGACGAGCTG<br>GAGAAGGCCATC<br>ACCACGCAGAAC<br>GTGAACACCAAG	HDR template for generating the knock-in mice	Single-strand DNA was used for zygote injection

	TGCATTACCATCC CCAGGTGGGGTC CCAGAGCGGCTG GGGGCGCACTG GCCTAGCCCCCT AGCGCGGCCCG GGTGGAGAGGGA GGGCGTGTGCGC GTGTGTGCGCGC GCCTGTAGGGGA CGGGGAGTGGGT GGAGGGACCCCA AACAAAACCTTC GGATTTGCCTAG CCTCAGAGACCT GGCTTCCG		

Complete content for Supplementary Datasets 1-12 can be downloaded here: 10.6084/m9.figshare.30992110

Supplementary Dataset 1: RNA-seq results for GDF9 stimulation in mouse granulosa cells.

Supplementary Dataset 2: Transcriptional Regulatory Relationships Unraveled by Sentence-based Text Mining (TRRUST) enrichment analysis results.

Supplementary Dataset 3: GO Enrichment results of the differentially expressed genes.

Supplementary Dataset 4: Gene overlapping results of the SMAD2-and HA-bound genes.

Supplementary Dataset 5: Gene overlapping results of the SMAD3-and PA-bound genes.

Supplementary Dataset 6: Peak annotations from the SMAD2 binding sites under GDF9 treatment condition.

Supplementary Dataset 7: Peak annotations from the SMAD3 binding sites under GDF9 treatment condition.

Supplementary Dataset 8: Peak annotations from the SMAD2 binding sites under Control treatment conditions.

Supplementary Dataset 9: Peak annotations from the SMAD3 binding sites under Control treatment conditions.

Supplementary Dataset 10: Peak annotations from the SMAD4 binding sites under Control treatment conditions.

Supplementary Dataset 11: Peak annotations from the SMAD4 binding sites under GDF9 treatment conditions.

Supplementary Dataset 12: Lists of core-direct target genes
